# Using social media listening to understand barriers to genomic medicine for those living with Ehlers–Danlos syndromes and hypermobility spectrum disorders

**DOI:** 10.1111/hex.13755

**Published:** 2023-04-16

**Authors:** Erika Kline, Amanda Leigh Garrett, Catherine Brownstein, Sonja Ziniel, Erica Payton, Aleah Goldin, Kathleen Hoffman, Judy Chandler, Shani Weber

**Affiliations:** ^1^ Inspire Arlington Virginia USA; ^2^ Division of Genetics and Genomics, Boston Children's Hospital, Department of Pediatrics, Harvard Medical School Boston Children's Hospital Boston Massachusetts USA; ^3^ University of Colorado School of Medicine, Department of Pediatrics, Section of Hospital Medicine Children's Hospital Colorado Anschutz Medical Campus Aurora Colorado USA; ^4^ The Ehlers‐Danlos Society 1732 New York New York USA

**Keywords:** Ehlers–Danlos syndromes, genomic medicine, health care access, hypermobility spectrum disorders, social media listening

## Abstract

**Introduction:**

Technological improvements alone have not led to the integration of genomic medicine across a broad range of diseases and populations. For genomic medicine to be successfully implemented across specialties and conditions, the challenges patients and caregivers experience need to be identified using a multi‐faceted understanding of the context in which these obstacles occur and how they are experienced. Individuals affected by rare conditions, like Ehlers–Danlos syndromes (EDS) and hypermobility spectrum disorders (HSD), express numerous challenges with accessing genomic medicine. Many patients living with rare diseases seek information and find comfort in online health communities.

**Methods:**

Social media conversations facilitated through online health communities are windows into patients' and caregivers' authentic experiences. To date, no other study has examined genomic medicine barriers by analysing the content of social media posts, yet the novel methodological approach of social media listening permits the analysis of virtual, organic conversations about lived experiences.

**Results/Conclusions:**

Using a modified social–ecological model, this study found that social–structural and interpersonal barriers most frequently impede access to genomic medicine for patients and caregivers living with EDS and HSD.

**Patient or Public Contribution:**

Data were retrieved through social media conversations facilitated through publicly accessible health communities through Inspire, an online health community. Social media listening permits the analysis of virtual, organic conversations about lived experiences.

## 1 INTRODUCTION

### 1.1 Access to health care

Policy shifts of the last decade have attempted to broaden access to quality health care and improve health outcomes with mixed results.^1^, ^2^ Under the Affordable Care Act insurance coverage has increased; however, in 2020, 28 million citizens (8.6% of the population) still lacked health insurance coverage at some point during the year.^3^ Unfortunately, any gap in coverage can negatively affect patient health outcomes. Even among insured individuals, barriers to accessing health care services remain, namely unreliable transportation, physician shortages and long wait times.^4^

Since the Institute of Medicine^5^ defined access to health care as ‘the timely use of personal health services to achieve the best health outcomes’, the definition has been explored and expanded, with both access to and utilisation of health care services as key dimensions.^6^ Patient‐centred care conceptualised access as a complex layering from personal through the structural dimensions, each with barriers and enabling factors on the pathway to quality care.^7^

### 1.2 Genomic medicine

Patients are eager to access the promise of genomic medicine.^8^ Genomic medicine, the use of patients' genetic information to inform clinical care (i.e., testing, treatment and counselling), has increased and will continue to do so in the coming decades.^9^ Genetic technologies have become more cost‐effective, accurate and efficient as health care institutions recognise their value in diagnosing diseases and guiding treatment recommendations^10^; yet clinical integration of genomic medicine has been surprisingly slow when compared to technological advancements.^11^

Implementing genomic medicine lags across a broad range of diseases and populations despite technological advances.^12^ Health care providers report feeling unprepared and unconfident in their ability to use genomic medicine in their practices.^13^ Similarly, patients report a limited understanding of the genetic tests available and difficulty interpreting their genetic results.^14^ Even among healthcare providers incorporating genomic medicine into regular clinical care, patients and caregivers can still encounter a myriad of obstacles, including high out‐of‐pocket costs, long wait times, language barriers, privacy concerns and discrimination, potentially further disrupting access to genetic testing, counselling and treatment.^9^, ^13^ For genomic medicine to be fully implemented across specialties and conditions, barriers patients and caregivers experience must be identified and addressed using a multi‐faceted understanding of the contexts in which these obstacles occur and how they are experienced.

### 1.3 Ehlers–Danlos syndromes and hypermobility spectrum disorders

**Figure 1:**
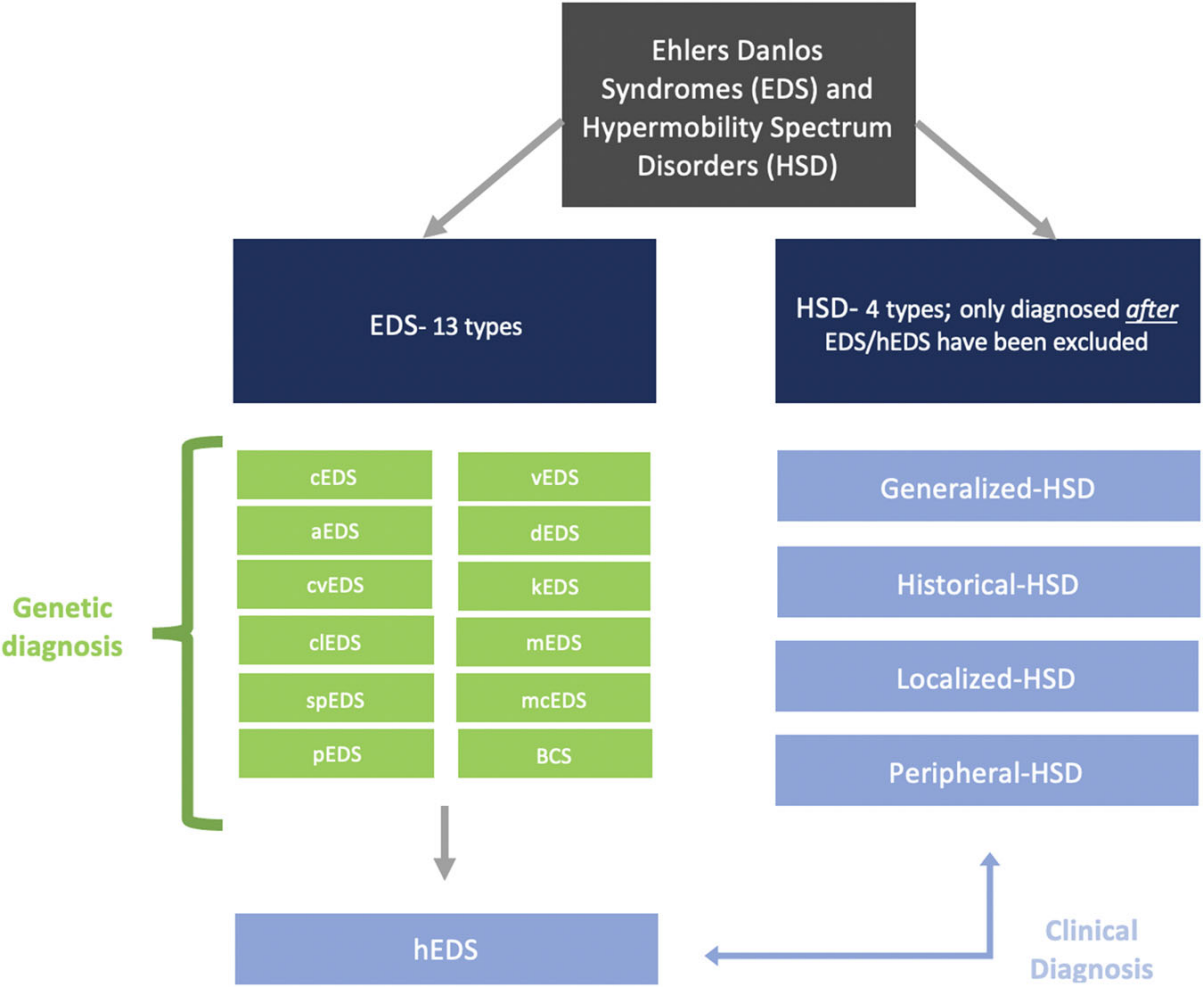
EDS and HSD types and diagnosis.

As a collection of rare diseases, Ehlers–Danlos syndromes (EDS) hypermobility spectrum disorders (HSD) are challenging conditions that are difficult to diagnose promptly and manage effectively.^15^ EDS (1:5000), a group of connective tissue disorders caused by genetically altered collagen is characterised by hypermobile joints, hyperextensible skin and/or chronic pain.^14^ With thirteen different subtypes and six classification groups based on pathogenic mechanisms, patients can present symptoms along a wide spectrum from mild to severe.^16^ HSD are a group of conditions with hypermobile joints, specifically the ability of joints to extend beyond a normal range of motion.^15^ HSD presents with similar joint pain symptoms as EDS but without connective tissue complications (see Figure 1 for EDS and HSD subtypes). HSD is more common than EDS, with estimates of the disorder as large as 1 in 500^16^ but clinicians agree that both EDS and HSD are underdiagnosed.^17^

EDS and HSD have discrete obstacles in their diagnostic odyssey. Once an individual is suspected of living with a form of EDS, their physical symptoms are examined along with family history, followed by genetic testing. One subtype of EDS, known as hypermobile EDS (hEDS), does not yet have identified genetic markers. Its diagnosis relies solely on clinical presentation.^18^ HSD do not have genetic underpinnings, leaving diagnosis to be exclusion‐based after EDS conditions have been omitted, including hEDS.^15^

Since EDS and HSD patients present across a range of complex symptoms and require a comparable commitment to disease management, both require interaction with myriad healthcare professionals (HCPs). The journey to an accurate diagnosis can be fraught for those with EDS or HSD. At one extreme, patients experience subtle external symptoms that may not immediately alert clinicians to their underlying condition; at the other extreme, patients may be accused of exaggerating symptom severity by clinicians.^16^, ^19^ Healthcare providers may assign psychological explanations for physical symptoms, call into question EDS/HSD patients' credibility and dignity and leave patients feeling ignored or belittled.^20^, ^21^

Repeated negative healthcare interactions can lead to patient mistrust of their physicians and a lack of proper long‐term medical treatment.^22^ Efforts to increase clinician literacy surrounding EDS/HSD have been made (e.g., ECHO).^23^, ^24^ Patients may have limited access to these knowledgeable providers and face numerous other barriers when seeking diagnosis and treatment. Therefore, many patients rely on social media to connect with other patients, caregivers and health professionals.

### 1.4 Social media and health

Currently, 70% of Americans report using at least one social media platform to connect with others, including members of various health communities.^25^ Patients and HCPs connect via social media to exchange information, find support and spread awareness.^26^ Social media is increasingly used as a health resource, at all stages of the disease journey, as patients and caregivers rely on this interface to share experiences, express emotions, seek information and obtain healthcare provider recommendations.^27^

Despite a significant increase in health‐centric social media use and technological advances in the field of genomics, research focussing on understanding implementation into clinical practice has lagged. Social media listening for health research has previously been used in other health‐centred investigations: e‐cigarette usage, gaps in health messaging, pharmacovigilance and beliefs and behaviours about medication concerns.^27^, ^28^, ^29^ To date, studies have not utilised a social media listening methodology to analyse the content of social media posts for virtual, organic conversations about lived experiences, particularly challenges in accessing genomic medicine.^30^, ^31^ The application of social media listening to understand the genomic medicine journey and health care experiences of individuals living with EDS/HSD has yet to be utilised. The aim of this study is to explore social media, particularly online health communities as a context, to uncover barriers to accessing genomic medicine among a population of those living with the rarified conditions of EDS/HSD.

### 1.5 Modified social–ecological model

The current study investigates barriers to accessing genomic medicine through a social–ecological framework. Traditionally, the social–ecological model (SEM) explains nested relationships among social–structural, interpersonal and individual levels of influence.^32^ The model accounts for the fact that individuals are influenced by interpersonal relationships and the broader societal context. The ability to access health resources is impacted by individual circumstances as well as the environment in which individuals are situated.^33^ Individual barriers are the knowledge, attitudes, behaviours and beliefs that shape an individual's autonomy to engage in health‐related decision‐making.^34^ Most frequently individual barriers are related to health literacy and in this study include a lack of information or misinformation about genomic medicine. Environmental factors are outside of direct patient control and include both interpersonal and structural barriers. Interpersonal barriers may refer to relationships with others such as friends, family and health care providers that influence behaviour.^34^ Difficulty getting a referral from a health care provider or disagreements among providers regarding appropriate diagnosis and management are several examples of interpersonal barriers. Although patients may seek access to genomic medicine, they can be obstructed by healthcare providers who influence healthcare decisions. While interpersonal barriers may be malleable for patients, social–structural barriers exist as the most removed barrier from patient control: societal norms, policies and practices that influence health‐related decision‐making.^34^, ^35^ Examples of social–structural barriers include long wait times to see geneticists, excessive travel times and high out‐of‐pocket costs. Addressing structural barriers, and to some extent, interpersonal barriers require institutional change.

In health research contexts, modified SEMs have expanded these three levels of influence to include functions of the product or technology (i.e., technological barriers) used in health care.^36^ The current model was specifically adapted from the infectious disease literature.^36^, ^37^ In the context of genomic medicine, technological barriers include any issues directly related to the software or hardware used to receive genomic medicine (e.g., inconclusive test results and difficulty accessing results on a website).

## 2 METHOD

Social media listening, or the study of online communication between individuals used to follow behaviours and interactions, is the overarching research methodology used in this study.^38^ Using social media data to analyse health issues including chronic illnesses and cancer has been shown to be an innovative and viable research approach.^28^ As a novel approach, social media listening allows researchers to examine barriers discussed via social media conversations, which may differ from those discussed in a room with a moderator or selected via a survey. As other studies have noted, this methodology captures opinions from patients and caregivers who might not choose to participate in a traditional study or who might answer differently in a formal research setting.^29^

**Figure 2:**
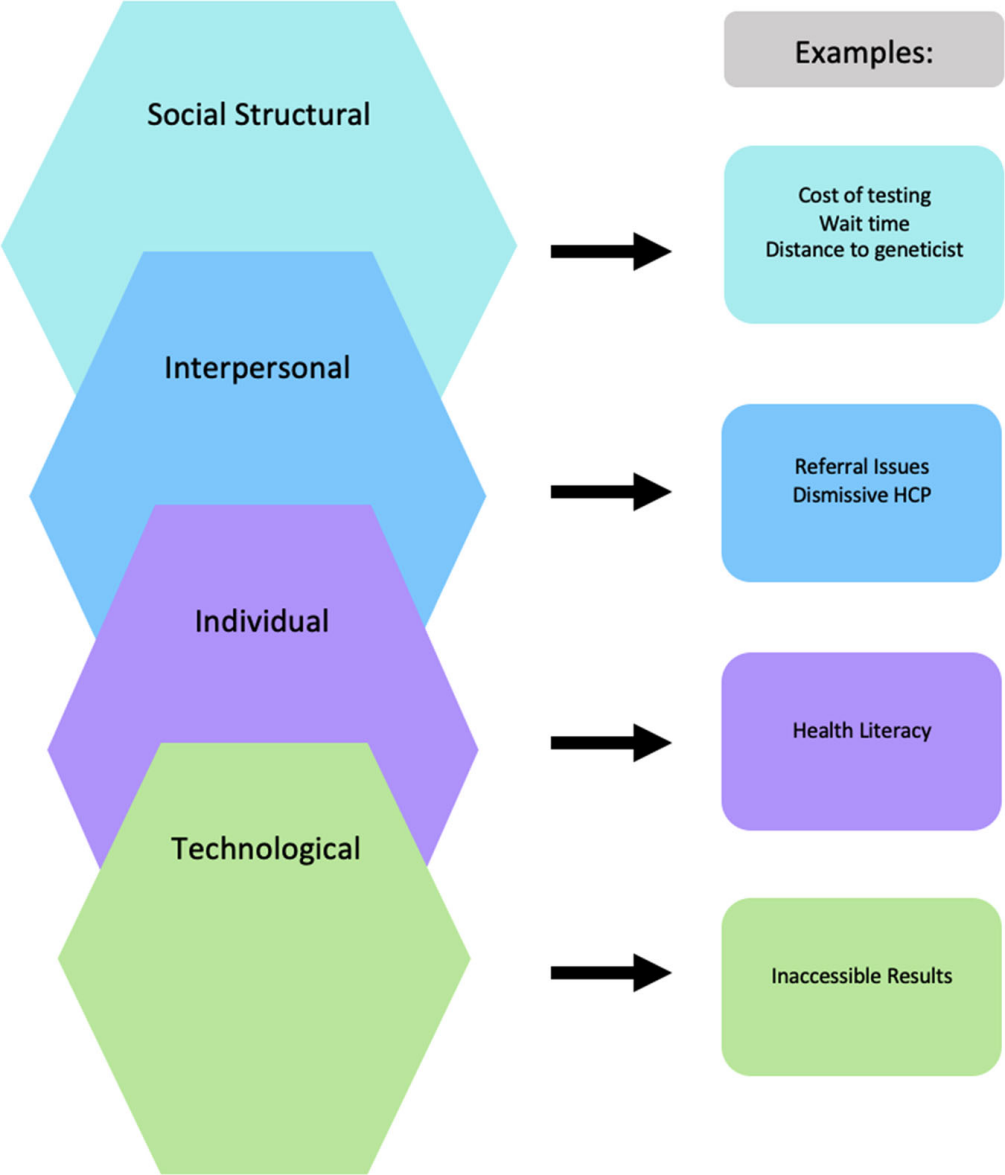
Modified social–ecological model.

Social media listening methodology was applied across a targeted sample of patient and caregiver conversations to identify barriers to genomic medicine access. Key barriers were identified and classified by examining the conversations within the modified SEM (i.e., social–structural, interpersonal, individual and technological) framework (see Figure 2).

### 2.1 Data extraction

The data set was extracted from Inspire, an online health community of over 2.5 million individuals with conditions and their caregivers (http://www.inspire.com). The Inspire community, with members residing in over 100 countries, represents over 3600 conditions including cancer, autoimmune diseases, rare diseases and other chronic conditions.

Inspire community members may anonymously engage with others with similar conditions through discussion posts and direct messaging. Inspire seeks to elevate research discoveries through its health communities. Deidentified community posts are available for secondary data analysis. Participants agree to have their posts/discussion comments utilised for research purposes upon registering for Inspire. As per Inspire's data privacy agreement, all quotations presented in this paper are public posts or posts obtained with member permission.

The data set was composed of social media posts from the EDS and HSD support community on Inspire in partnership with the Ehlers–Danlos Society. The extracted discussion posts and replies contain mentions of genomic medicine. All posts contain one or more agreed‐upon keywords or TextRazor tags (see Tables 1 and 2). Additionally, all posts were extracted from a 2‐year timeframe (10/01/19‐09/30/21) to ensure the timeliness of the data, given that barriers to accessing health care services can change over time. After removing duplicate posts, the data set contained 1949 unique posts from 232 members of the EDS and HSD community.

**Table 1:** Keywords.

gene^a^ personalised medicine | precision medicine | targeted treatment tumour testing liquid biopsy (saliva test | or saliva testing)!(covid‐19 | coronavirus | covid) biomarker^a^ CRISPR molecular mutation^a^ insertion | deletion | translocation| recombinant sequencing | NGS exome DNA|RNA pharmacogenomics CMA chromosome^a^ microarray 23andme|23 and me ambry foundation medicine | foundationmed | foundationOne | foundation one Miraca helix GeneDx Genomind illumina invitae natera progenity tellmeGen macrogen guardant^a^

*Note*: Keywords were used to extract Inspire member posts related to genomic medicine.

^a^ A search term was broadened to find all words that started with the same letters.

**Table 2:** TextRazor tags.

Biomarker | BiomarkerDiscovery | CancerBiomarker ChromosomeAnalysis | ChromosomeAbnormality DNA|DNASequencing | DNATest | MitochondrialDNA EpigeneticCode | Epigenetics Gene | GeneEditing | GeneExpressionProfiling | GeneExpressionProfilinginCancer GeneticCode | GeneticCounseling | GeneticPredisposition | Geneticist | GeneticTesting, GeneticDisorder | CancerGenetics Genome | GenomicDNA | Metagenomics | TheCancerGenomeAtlas | GenomeProject, HumanGenomeProject | CancerGenomeProject | HumanGenome Heredity HumanGenetics | MedicalGenetics MolecularAnalysis | MolecularDiagnostics | MolecularGenetics | MolecularImaging, MolecularMarker | MolecularMedicine | MolecularPathology MutationTesting PersonalizedMedicine | PrecisionMedicine Pharmacogenomics Sequencing | WholeGenomeSequencing | ExomeSequencing FoundationMedicine | Ambry | GeneDx | MyriadGenetics | Natera | Helix | Macrogen SalivaHormoneTesting

*Note*: TextRazor is a natural language processing API that provides automatic topic detection and tagging of unstructured content. The following TextRazor tags were used to extract Inspire member posts related to genomic medicine.

### 2.2 Data analysis

Once the relevant posts were identified, researchers independently coded the data using NVivo 12 Pro, a qualitative data analysis software.^39^ The coding team was composed of two researchers, including E. K., an applied social scientist and A. G., a qualitative researcher. The authors undertook a deductive coding approach, using the modified social–ecological framework. An extensive codebook was developed to operationalise each of the applied terms. The coders completed two rounds of coding: The first round identified posts that mentioned experienced barriers to genomic medicine using Scheppers^40^ definition of barriers, ‘anything that restricts the use of health services by making it more difficult for some individuals to access, use, or benefit from care’. The second round of coding catalogued the author and the level of influence of the barrier, using the modified SEM. The individual experiencing the barrier to genomic medicine was coded as the author. For example, if a post mentioned that both a mother and child had the same condition but only the child experienced a long wait time for genetic testing, then the author was only coded as ‘caregiver’. The levels of influence coded in the second round using the modified SEM were sociostructural, interpersonal, individual, technological and not enough information (see Table 3).

**Table 3:** Code definitions.

Round of coding	Code	Code definition
Round 1	Experienced barrier	An obstacle that a patient experienced themselves or that a loved one experienced when attempting to gain access to genomic medicine.
Round 2	Technological barrier	Issues directly related to the software or hardware currently used to receive genomic medicine.^36^
	Individual barrier	Knowledge, attitudes and beliefs that shape an individual's autonomy to engage in health‐related decision‐making.^34^
	Interpersonal barrier	Relationships such as family, friends, neighbours, health care providers and others that directly influence health and health behaviours.^34^
	Social‐structural barrier	Societal norms, conditions, local state or national laws, policies and practices; relationships between organisations and groups; and geographical/political regions that have an influence on health and health‐related decision‐making.^34^
	Not enough information	Not enough context clues in the discussion or reply to identify the type of barrier.^34^, ^35^

The coders independently coded 28% of the data (the first 540 posts) for the first round of coding and achieved an interrater reliability (IRR) of *κ* = 0.80. Kappa statistics were chosen to report as the coefficient takes into account agreement among coders due to random chance. After posts were identified as containing a barrier, the coders had a one in five chance (20%) of selecting the same barrier classification simply by chance, therefore a Kappa statistic provides a more conservative estimate of IRR. NVivo software calculates both the Kappa statistic and percent agreement among coders. All discrepancies observed in the first 540 posts were discussed among both coders until an agreement was reached. The remaining 1409 posts were distributed among the coders and were systematically checked for moderate to strong reliability ratings. The second round of coding consisted of the identification of the author and levels of influence using the modified SEM. Both researchers independently coded 33% of the data (the first 115 posts) for the second round of coding and achieved IRR of *κ* = 0.83. Descriptive statistics were used to summarise the percentage of posts by members.

## 3 RESULTS

### 3.1 Experiences

From our data set of 1949 unique posts, referencing genomic medicine from Inspire's EDS and HSD community, 345 unique posts were identified as referencing barriers when accessing genomic medicine. The majority of barriers experienced within the EDS and HSD community were at the social–structural level 50% (174/345) and the interpersonal level 33% (115/345). Twenty‐three percent (81/345) of the barriers were at the individual level. Of these 345 posts that mentioned barriers, only 4% (14/345) contained references to technological barriers. Identification of the level of influence was not possible for 3% (12/345) of barriers due to the lack of context in those particular social media posts (see Table 4). In some posts, 15% (51/345) mentioned two or more levels of influence in the same post: social–structural and interpersonal, 5% (18/345) and social–structural and individual 6% (19/345), co‐occurred most frequently.

**Table 4:** Posts coded by author and level of experienced barrier.

Author	Total	Barriers				
		Social–structural	Interpersonal	Individual	Technological	Not enough information
		*n* (%)	*n* (%)	*n* (%)	*n* (%)	*n* (%)
Patient	296	154	99	65	11	11
Caregiver	38	17	16	9	2	1
Health care provider	2	1	0	0	1	0
Unknown–other	9	2	0	7	0	0
Total	345	174 (50%)	115 (33%)	81 (23%)	14 (4%)	12 (3%)

*Note*: Totals for coded barriers are greater than 345 because each post could be coded for multiple barriers.

In total, the 345 posts from Inspire's EDS and HSD community containing experienced barriers were authored by 232 patients, caregivers, healthcare providers or unknown/other authors with the majority (77%) of Inspire members writing a single post about their difficulty accessing genomic medicine (see Table 5). All quotations presented in this paper were public posts or posts obtained with member permission.

**Table 5:** Number of posts by EDS/HSD members.

Number of posts	Number of members
1 Post	179
2 Posts	27
3 Posts	13
4 Posts	4
5 Posts	3
6 Posts	3
7 Posts	0
8 Posts	3

*Note*: *N* = 232 members.

### 3.2 Social–structural

Social–structural barriers are societal norms, conditions, local state or national laws, policies and practices; relationships between organisations and groups; and geographical/political regions that influence health and health‐related decision‐making.^34^, ^35^ Most barriers impeding genomic medicine access stray beyond the patient, caregiver and healthcare providers' ability to directly control. Patients reveal a variety of barriers: frustration over long wait times to see geneticists, extensive travel time to appointments due to distance to see knowledgeable geneticists, insurance companies' refusal to pay for initial or additional genetic testing, and high copayments for genetic testing. Other examples of barriers to genomic medicine include demonstrating a family history of EDS, having a prior diagnosis of EDS, or requiring parent testing before child testing.

**Wait time and distance to a geneticist**

I was just diagnosed with [hypermobile] EDS this week. I have been thinking I've had it for 2 years but had to find a doctor to listen, then had to get a referral to a hospital 3 and a half hours away to see the geneticist. (Member 207)

I had never heard of EDS and waited a year to see a geneticist once I came across it. (Member 252)

**Out‐of‐pocket cost**

I have already been to just about every specialist you can think of over this last 5 years because my doctor started with them first. Genetics was last and it cost me out of pocket. (Member 123)

Unfortunately, because it's so expensive and insurance does not want to pay for it, many zebras, like myself, go without genetic testing at all. (Member 673)

The preponderance of social–structural barriers within patient and caregiver discussions point to serious systemic issues in obtaining genomic medicine for those living with EDS/HSD. A lack of geneticist offices throughout the country (particularly in rural areas) and poor insurance coverage for testing continue to be described by patients and caregivers resulting in diagnosis and treatment delays. Health insurance often does not cover genomic medicine testing, leading to high out‐of‐pocket costs, financial stress or foregoing care.

### 3.3 Interpersonal

Interpersonal relationships refer to the influence individuals with close ties (like family, friends, neighbours and trusted health care providers) have on health and health behaviours. Those living with EDS and HSD in our study frequently report experiencing interpersonal barriers when seeking access to genomic medicine. Specific examples include the following: an inability to get an appropriate genetic test, disagreement among health care providers about diagnosis, dismissive and/or rude health care providers, lack of treatment options, geneticist referral issues and finally, healthcare providers not taking on new patients.

**Dismissive responses from health care practitioners**

I also had a bad experience with Dr. [Name] He totally dismissed me (he wouldn't even perform any genetic testing). (Member 575)

My daughter and I went to a geneticist at a large hospital system in [location]. There is no genetic marker for [hypermobile] EDS. They left us high and dry and refused to confirm our existing diagnosis regardless of all of the party tricks we were able to show. There was no follow‐up offered except to refer us to an Acupuncturist. It was heartbreaking. (Member 622)

These comments each illustrate a lack of access to quality care, leaving those with EDS with unmet expectations. Even when explicitly requesting genomic medicine, some families report healthcare provider refusal.

**Referral issues**

When I mentioned EDS to the doctor they assigned me to she said ‘we don't have time to get into that right now’, but her face told me she had no idea what I was talking about. I do not have much faith she will refer me to a geneticist. (Member 695)

Interactions showcasing barriers to genetic medicine were common between patients and caregivers and those in health care professions. These experiences highlight the struggle of patients to be understood for a complex and less common condition. EDS and HSD patients and caregivers seek validation, confirmation, respect and follow‐up guidance in their interpersonal communication regarding genetics from their HCPs.

### 3.4 Individual

Individual barriers are the knowledge, attitudes, behaviours and beliefs that shape an individual's autonomy when engaging in health‐related decision‐making.^34^ Those living with EDS/HSD can experience guilt about potentially passing on a genetic condition, which hinders their willingness to access genomic medicine. Health literacy and genetic literacy act as individual barriers to genomic medicine access. The majority of individual barriers centre around the patient or caregiver's lack of information or misinformation about genomic medicine. Other individual barriers include concerns about having a diagnosis on their medical files (lack of trust for insurance companies/doctors), worry about age (considering themselves or a loved one too young/too old to get tested), confusion about how variants of certain genes impact whether they have the condition, and finally seeking recommendations for geneticists, and comorbidities.

**Health literacy**

Hypermobility alone is not enough for an hEDS diagnosis. However, all the geneticist is qualified to do is diagnose. Do not be disheartened that the geneticist was not helpful about treatment—they would not have been helpful about that even if hEDS was diagnosed. (Member 363)

My son was diagnosed with hEDS about 2 years ago and I was told it was genetic. I haven't had the opportunity to take him or myself to genetics yet due to all of his other medical issues such as Autism (ASD). Being a single mom makes it hard to be in 100 places at once. What will genetics help with? What should I watch out for in my son besides what I have already seen? (Member 314)

Patient and caregiver knowledge, attitudes and beliefs about the purpose and benefits of genetic medicine drive care and treatment seeking. Decision‐making without complete information on the roles of various health care proffesionals is common. It is not common knowledge that geneticists are qualified to both diagnose conditions and provide treatment and counselling, which often results in delaying or forgoing genetic testing.

### 3.5 Technological

Technological barriers to genomic medicine include software or hardware issues. For example, these barriers may be methodological (in screening for genetic conditions) or issues with the electronic health record system (i.e., navigability, clarity of results).

Although patients, caregivers and occasionally health care providers in this study reported experiencing relatively few technological barriers in accessing genomic medicine, consistent concerns about test accuracy, inconclusive results and uninterpretable results persisted. Technological barriers mentioned were often similar to the barriers experienced by this EDS patient:

**Inaccessible results**

Does anyone know how to interpret raw genetic data to assess for different types of EDS? I searched online for specific SNPs to look up, but all I could find is the genes where different types of EDS stem from, but nothing about how to specifically assess those genes. Unfortunately, while there is very clear instructions on how to use your raw data to assess yourself for many diseases, I can' find any similar instructions for EDS. (Member 311)

While consumer genetic tests are becoming more widely available and more affordable to patients, specific EDS results are not easily accessible via the internet. Even when test results are available, without healthcare provider guidance, many patients are left with genetic results that they are not able to understand or utilise to improve their health. Often the user interface and navigation associated with patient‐facing genome medicine software are not intuitive, which can lead to problems in the patient interpretability of results.

## 4 DISCUSSION

Using a modified SEM as a framework, social media data from those living with EDS/HSD were explored to better understand how barriers to genomic medicine were described. To date, this study is among the first to catalogue barriers to accessing genomic medicine that patients, caregivers and occasionally health care providers discuss organically online. It is also one of the first studies to situate these barriers within a theoretical framework. The modified SEM framework, long employed to promote health outcomes, was useful to identify obstacles influencing care that operate in symbiotic social–structural, interpersonal, individual and technological domains. The advantage of using this model was that it provided a dynamic and integrative framework to conceptualise multiple levels of influence and reciprocal interdependencies.^37^, ^41^, ^42^ The nature of the relationship between levels of constructs helps scholars transcend discussions focussed too narrowly on one level of influence while permitting identification of the levels that patients and caregivers most frequently and organically mention when trying to access genomic medicine.

Many patients primarily use social media for health communication to increase health literacy and gain information about their health or the health of a loved one, therefore^25^, ^43^ patients would theoretically be more likely to report individual barriers because they are coming to the site looking for information (i.e., increasing health literacy). However, an important finding of this research was that social–structural barriers and interpersonal barriers, not individual barriers, were written about most frequently with rates 10%–47% higher than other levels of influence. For example, patients and caregivers expressed barriers at the social–structural level, including long wait times and long distances to geneticists. Removing social–structural barriers requires institutional changes like the adoption of more virtual appointments by healthcare providers to increase access and decrease wait times.^44^ This finding highlights the importance of addressing patient barriers at multiple levels of influence, particularly in situations where patients have little to no direct control over outcomes.

The barriers observed within this study largely align with other studies that have documented barriers to genomic medicine utilisation among patients and caregivers.^12^, ^13^, ^45^, ^46^ Within the literature, many of the obstacles patients face when accessing genomic medicine are structural, such as high out‐of‐pocket costs, long wait times and long distances to the nearest geneticist with availability.^6^, ^9^, ^12^, ^13^ Concerns about patients bearing the financial cost of genomic medicine are widely recognised, with many physicians expressing uncertainty about whether genomic testing can be implemented into regular clinical care due to the lack of reimbursement options outside of a research setting.^47^ Even where genomic medicine is more readily available, affordability can still remain an insurmountable structural barrier.

Interpersonal issues between those living with EDS/HSD and those working in health care are major obstacles to accessing genomic medicine. Patients report experiencing interpersonal problems getting referrals, seeing a geneticist who is informed about their particular condition, receiving a variety of treatment options, and receiving conflicting information from different healthcare providers. Largely, the aforementioned interpersonal barriers patients report could be attributed to a lack of knowledge and preparedness by physicians.^13^ Specific to individuals with EDS and HSD, patients have consistently reported being ignored and belittled by HCPs and assigned psychological and/or psychiatric explanations for their symptoms.^20^ Patients who feel that their symptoms and conditions are not being validated may be more likely to have negative interpersonal interactions with health care providers. These negative interpersonal interactions denote a lack of appropriateness in access to care offered by healthcare providers. Unreliable interactions do not denote quality health care or provide patients with continuity of care.^6^ Our study confirms a significant challenge for patients with this rare and often invisible disease—being believed. Genomic medicine and its advances can provide an important confirmation of the patient's experience and diagnosis. Without it, these patients will continue to be under‐treated.

Validating symptoms for individuals living with EDS/HSD would be an important step toward restoring patient confidence in health care while helping to break down another barrier, acceptance of genomic medicine information. Individuals possess varying beliefs about health, health literacy and willingness to agree to health services.^6^ Individuals may not fully appreciate the benefits of genetic testing/counselling and therefore do not choose to pursue this path. As Levesque et al.^6^ suggest, self‐efficacy and motivation to engage in health care are necessary for accessibility. Instilling genomic testing into the diagnostic process and guiding patients to understand its benefits will lead to increased genomic health literacy and improved patient confidence, ultimately making online sharing of information more accurate while reducing the spread of misleading patient conversations.

Many look to online communities for answers. Low health literacy can lead to the unintentional spread of misinformation in virtual spaces. Public posts with inaccurate information, as seen in one example we provided, can be very harmful. As patients exchange information with one another, that misinformation can create a ripple effect leading to more misconceptions about genomic medicine among other health community members. Providing accurate information, ideally from a HCP, could change opinions about genomic medicine and reduce the spread of misinformation.^48^

Genomic literacy remains low, indicating that while consumers have broad access to information about their genetics, they do not have access to the expertise to understand their test results.^9^ The technical nature of genetic laboratory results often leads to communication breakdowns between providers and patients resulting in misinterpretations of the laboratory results. Using comprehensive electronic health record systems and revising current genomic test reports to be more patient‐friendly could improve accessibility for patients and reduce technical barriers.^49^ As genomic medicine continues to gain in popularity, patients will voice concerns over technological barriers to information, paving the way for improvements in software tools and the availability of helpful patient educational resources.

## 5 LIMITATIONS

Although we were able to utilise a large sample of EDS and HSD patients via Inspire, one limitation of our study is inherent to our sample. Researchers have routinely documented the disproportionate representation of users on social media platforms. Social media users are more likely to be white, female, older, more educated and higher‐income earners as compared to the general population.^25^ Individuals who use social media for health purposes mirror similar demographics with most users skewing older, more educated and earning higher incomes.^50^ Historically, Inspire, like most online health communities, has not systematically collected information on the demographic characteristics of its members. Previous research has shown that social‐structural barriers more frequently impact self‐identifying Blacks and Latinx compared to other racial groups.^44^, ^51^ Unfortunately, the researchers were unable to assess levels of experienced barriers by race/ethnicity for this study. Additionally, EDS/HSD diagnoses cannot be confirmed as members self‐report information about their diagnosis and treatment.

A second limitation of this study is the type of posts on social media health platforms. According to the negativity bias, online health community members tend to compose posts that are negatively balanced to garner attention and social support.^52^, ^53^, ^54^ Therefore, members are more likely to post about extremely salient negative interactions they have experienced, for example, a rude and/or dismissive geneticist (an interpersonal barrier) versus a more moderate negative interaction like technical difficulties with patient‐facing genome software (a technological barrier). The negativity bias may have influenced the base rates obtained for each of the levels of influence, specifically an underreporting of technological and individual barriers and an overreporting of interpersonal barriers.

## 6 CONCLUSION

Using a modified SEM combined with social listening methodology, posts were analysed from those with a self‐reported diagnosis of EDS and HSD, to learn about their experienced barriers when accessing genomic medicine. Overall, patients reported experiencing social‐structural barriers most frequently, followed by interpersonal, individual and technological barriers. Efforts to systematically and sustainably integrate genomic medicine into clinical practices must address factors at all levels of the social–ecological framework. Patients disproportionally faced barriers outside their immediate control, including social–structural, interpersonal and technological barriers. Creating improved access to genomic medicine requires policy and institutional changes to ensure patients receive better and more comprehensive genetic diagnosis and treatment.

Applying the novel social listening methodology helped identify barriers that might not as readily emerge in interviews, focus groups or surveys without prompting participants. For example, many patients might not be aware that the wait times and/or cost of out‐of‐pocket testing they face are structural barriers. Patients routinely were relieved by only having to wait a ‘few months’ to see a geneticist and only pay a ‘few hundred dollars’ for testing. Asking participants more broadly if they faced any structural barriers in a survey question might have prompted users to say that they had not encountered any structural barriers; whereas, using the social listening methodology allowed researchers to catalogue barriers that patients and caregivers might not have identified themselves as barriers. The unstructured, real‐time and impromptu nature of social listening conversations uncovers sentiment often absent in other methodologies. Future studies should continue to catalogue mentions of genomic medicine barriers on other social media platforms, such as Twitter and Reddit, using the modified social–ecological framework. More research on specific barriers experienced by patients and caregivers is warranted, especially in the rare disease space where patients regularly feel dismissed and ignored.^45^, ^46^ Obstacles to genomic medicine experienced by persons with intersecting vulnerabilities, such as patients with rare diseases who identify as racial and ethnic minorities, merit further research as well.^12^

## AUTHOR CONTRIBUTIONS

Erika Kline and Aleah Goldin conducted social listening and produced the first draft of the manuscript. Catherine Brownstein is the Principle Investigator for the (NIH) RFA‐HG‐20‐037 at Boston Children's Hospital and oversaw all aspects of the project and along with Kathleen Hoffman approved social listening terms, codebook, research, wrote and edited the manuscript. Amanda Leigh Garrett managed the grant, wrote and edited the manuscript, Erica Payton wrote and edited the manuscript and Sonja Ziniel edited the manuscript. Shani Weber edited and provided guidance on Ehlers–Danlos syndromes and hypermobility spectrum disorders.

## CONFLICT OF INTERESTS STATEMENT

Catherine Brownstein works at Boston Children's Hospital, and Sonja Ziniel works at Colorado Children's Hospital. Amanda Leigh Garrett, Erika Kline, Erica Payton, Kathleen Hoffman and Aleah Goldin are former or current employees of Inspire.

## ETHICS STATEMENT

The New England Institutional Review Board reviewed this research project and found it to be exempt. All data were evaluated without the knowledge of the identity of those involved.

## Data Availability

Data that support the findings of this study are available from the corresponding author upon reasonable request.
